# Relationship between exercise intensity and stress levels among U.S. medical students

**DOI:** 10.1080/10872981.2022.2027651

**Published:** 2022-01-20

**Authors:** Richard K. Leuchter, Margaret L. Stuber, Austin L. McDonald, Daniel M. Croymans

**Affiliations:** a Department of Medicine, David Geffen School of Medicine, University of California, Los Angeles, CA, USA; b Department of Psychiatry and Biobehavioral Sciences, University of California at Los Angeles, Los Angeles, CA, USA; c Department of Emergency Medicine, Indiana University School of Medicine, Indianapolis, IN, USA

**Keywords:** Physical activity, exercise intensity, medical student, well-being, distress

## Abstract

**Background:**

Physical activity may protect the mental health of medical students, yet it is unknown which types and intensities of physical activity have the greatest potential to improve medical student well-being.

**Objective:**

We characterize the relationship between exercise intensity and stress levels of U.S. medical students, thereby informing the design of future well-being interventions.

**Design:**

Two cross-sectional validated surveys assessing stress and physical activity were administered one year apart at the David Geffen School of Medicine at UCLA. A total of 1,046 out of 1,392 medical students responded (75%). An ordered logistic regression was used to determine the association between stress and each level of exercise intensity (inactivity, moderate-activity, and health-enhancing physical activity [HEPA]). These exercise intensity groupings were compared to the CDC guidelines for aerobic exercise.

**Results:**

While achieving either moderate-activity or HEPA is compliant with the CDC guidelines for aerobic exercise, the additional intensity of exercise required to achieve HEPA was associated with a 26% increase in the probability of being in the lowest stress quartile and a 22% decrease in the probability of being in the highest stress quartile. Medical student physical activity levels were on-par with the national average per the CDC exercise guidelines (65% vs. 58%), but medical student HEPA levels were significantly lower than the national average (27% vs. 64%; OR 0.21; 95% CI 0.12–0.37).

**Conclusions:**

There is a large disparity in rates of the highest intensity physical activity (HEPA) between medical students and the age-adjusted national average, which has previously been overlooked by the binary CDC exercise guidelines. The fact that HEPA levels are not optimized and more strongly associated with lower stress levels relative to less intense forms of exercise makes it a promising new target for future well-being interventions among medical trainees.

## Introduction

Psychological distress, understood as a spectrum of presentations ranging from syndromes such as burnout to formal psychiatric disorders such as depression[1], is widely recognized as deleterious to the professional and personal lives of medical students [2–11]. Because lack of autonomy, low status in the medical hierarchy, and intense physical, emotional, and intellectual demands all contribute to distress, junior trainees (including medical students) who have yet to develop personalized coping mechanisms are particularly susceptible to distress [5,8,12,13]. Distress experienced during training can have longstanding effects and is associated with serious consequences including alcohol use disorders and higher rates of suicide [2,14–16].

Research has demonstrated an inverse relationship between exercise and distress [13,17–20] and even suggested that physical activity may protect medical trainees against mental illness [21]. While the mechanisms underlying this relationship are incompletely understood, they are likely bidirectional and a combination of physiological (e.g., cardiorespiratory fitness), biochemical (e.g., hormones), and psychosocial (e.g., physical self-efficacy and self-esteem and social networking) factors [22,23]. Despite the benefits of exercise, multiple recent reviews have disclosed that no wellness interventions have prescribed exercise to successfully decrease distress among U.S. medical students [16,24–26]. This fact highlights the need to first understand which types and intensities of exercise have the greatest potential to reduce distress among medical students. As an important component of psychological distress and an upstream mediator of depressive symptoms and burnout among healthcare workers [27,28], in this study we focused on perceived stress and its relationship to exercise among medical students.

Most investigations into medical trainee physical activity have applied national guidelines developed by the Centers for Disease Control and Prevention (CDC) [29], and have demonstrated that U.S. medical students are comparably active to the national average [18,21,30–32]. These guidelines characterize individuals as either compliant or noncompliant based on the total weekly minutes of activity in which they engage. This binary classification scheme overlooks the fact that while all exercise has physical health benefits, greater intensities of exercise confer greater benefits [33,34]. The World Health Organization has adopted an instrument called the International Physical Activity Questionnaire (IPAQ) that addresses this problem by further breaking down the compliant category into moderate physical activity and a higher-intensity physical activity called health-enhancing physical activity (HEPA) [35–37]. While HEPA offers more positive effects on physical health than do lower intensities of exercise [37,38], it is unknown if the relative benefits of HEPA extend to the perceived stress of U.S. medical students.

We hypothesized that among medical students, HEPA would exhibit a stronger inverse relationship with stress than would moderate physical activity. We further hypothesized that medical students would have similar levels of physical activity compared to the national average according to the CDC exercise guidelines but that the IPAQ guidelines would reveal that medical students engage in comparatively less HEPA than the national average. To test these hypotheses, we conducted two cross-sectional surveys that measured levels of HEPA, moderate physical activity, and inactivity and analyzed each tier in relation to perceived stress levels.

## Methods

### Participants and data collection

All medical students at the David Geffen School of Medicine at UCLA were eligible to participate. Surveys were anonymous and administered by pen and paper at mandatory class-wide meetings in the winter of 2013 and repeated in the winter of 2014. The populations studied at both times were sampled from the same class-wide meetings, and no a-priori power analysis was conducted, given that it would not have affected data collection since the goal was to capture as many medical students as possible. The total number of students present at these meetings was recorded and used to calculate response rates. Class meetings for first (MS1)- and second (MS2)-year students were at least 2 weeks away from any exams, and class meetings for third (MS3) and fourth (MS4) year students were in between blocks of clinical rotations.

Students were given 15 minutes to complete the surveys, after which they were collected and coded by hand in Microsoft Excel (Microsoft, Redmond, WA, U.S.). An independent reviewer cross-checked every input against the original documents for accuracy. The surveys were voluntary and anonymous, and no incentive for participation was offered. The UCLA Institutional Review Board certified the study as exempt prior to enrollment of students.

### Study measures

In addition to basic demographic information, the survey assessed generalized perceived stress, physical activity, and amount of sleep. Sleep was included in the logistic regressions in order to control for the fact that the volume and quality of sleep is itself associated with mental health and exercise levels [1,7,11,17].

The Perceived Stress Scale (PSS) is a 10-item instrument that quantifies the degree to which situations in life are perceived as stressful [39]. The PSS (available at https://www.sprc.org/system/files/private/event-training/Penn%20College%20-%20Perceived%20Stress%20Scale.pdf) includes the question stem ‘In the last month, how often have you felt … ’, followed by such items as *that difficulties were piling up so high that you could not overcome them*, and *that you could not cope with all the things that you had to*. It has an internal reliability (measured by Cronbach’s alpha) of 0.91 and has been validated in multiple national samples within the U.S. [40].

The International Physical Activity Questionnaire Short Form (IPAQ-SF) measures frequency and duration of different intensities of exercise [35]. Within the U.S., the IPAQ has a reliability (measured by Spearman’s reliability coefficient) ranging from 0.66 to 0.89, is validated in over 20 countries, and was adopted by the WHO to categorize physical activity into low activity, moderate activity, or HEPA (www.ipaq.ki.se) [37]. We compared exercise levels in medical students to an age-adjusted national average, given that age has been shown to be the strongest predictor of exercise [36]. The most recent national survey conducted using the IPAQ is from 2002 to 2004, at which time the proportionate breakdown of exercise intensities among U.S. males 18–39-year-olds was 11% inactive (also referred to as low-activity), 16% moderately active, and 72% HEPA-active, and U.S. females 18–39-year-old was 18% inactive, 26% moderately active, 56% HEPA-active (average 15% inactive, 21% moderately active, and 64% HEPA-active) [36].

The IPAQ defines HEPA as vigorous-intensity activity ≥3 days and accumulating ≥1500 MET-minutes/week or ≥7 days of any combination of walking or moderate-, or vigorous-intensity activities accumulating ≥3000 MET-minutes/week. Moderate physical activity is defined as ≥3 days of vigorous activity of at least 20 minutes/day, ≥5 days of moderate-intensity activity and/or walking ≥30 minutes/day, or ≥5 days of any combination of walking or moderate- or vigorous-intensity activities achieving ≥600 MET-minutes/week. Inactivity is defined as any level of activity not meeting HEPA or moderate physical activity. MET-minutes/week are the product of minutes of activity/day, days of activity/week, and one of the following constants based on exercise intensity: 3.3 for walking, 4.0 for moderate intensity, and 8.0 for vigorous intensity. For example, engaging in some combination of heavy lifting, aerobics, and fast bicycling for 70 minutes three times weekly would qualify as HEPA, whereas engaging in the same activity for 30 minutes five times weekly would qualify as moderate physical activity. These constants are obtained from exercise physiology studies quantifying the volume of oxygen utilized during activities of varying intensity [41].

The CDC aerobic exercise guidelines classify someone as compliant with guidelines if they attain weekly totals of ≥150 minutes of moderate activity, ≥75 minutes of vigorous activity, or some combination thereof [29]. As an example, anyone engaging in some combination of heavy lifting, aerobics, and fast bicycling for 75 minutes once weekly would be compliant with CDC guidelines. The National Center for Health Statistics reported that 58% of 18–44-year-olds in the U.S. were compliant with the CDC guidelines in 2014 [42].

Sleep was quantified using the fourth component of the Pittsburgh Sleep Quality Index (http://www.opapc.com/uploads/documents/PSQI.pdf), which determines how many hours of sleep an individual gets per night and has an internal reliability (measured by Cronbach’s alpha) of 0.83 [43].

### Statistical analysis

The primary analysis involved descriptive summary statistics to obtain means and proportions of demographic characteristics, exercise, and stress. The PSS scores were converted into quartile groups per common practice such that odds ratios and predicted probabilities could be calculated [44–47]. Given the ordinal nature of tiers of stress, an ordered logistic regression was performed with PSS quartile group as the dependent variable and IPAQ group, gender, amount of sleep, and year in medical school as the independent variables. For ease of understanding, the odds ratios from this regression were transformed into predicted probabilities (e.g., the probability of being in the highest stress quartile at each intensity of exercise) for each IPAQ group at the highest and lowest quartiles of stress while holding all other variables at their means.

To compare the overall proportionate breakdown of exercise intensity – i.e., inactivity, moderate activity, and HEPA – between medical students at each level of training and the national average, we used chi-squared tests. Odds ratios were calculated from these by the Baptista–Pike method to compare the frequencies of specific exercise intensities between medical students and the national average (i.e., percentage of individuals achieving HEPA in medical school vs. the national average).

We performed a one-way ANOVA to compare PSS scores between years of training. A post-hoc analysis of this ANOVA to determine specific group effects (among each level of medical student training) was done with Sidak’s multiple comparisons test, given that it makes consistently conservative estimates and performs well in the context of unequal sample sizes and unequal variance [48]. All analyses were performed using Stata/IC 16.1 (StataCorp LP, College Station, TX, U.S.).

## Results

Of the 1,392 eligible respondents over the 2 years of survey administration, 1,046 (75%) completed the questionnaire. Basic demographics, reported stress, and exercise intensities are reported in Table 1. According to school-wide demographic data, the 25% of non-responders were not statistically different from the responders for the reported demographics.

**Table 1.** Characteristics of study population including perceived stress and breakdown of physical activity intensity

Year in school	Number (response %)		Mean age (years)		% Female		PSS (95% CI)		IPAQ PA breakdown^a^ (% HEPA, moderate, inactive)		CDC PA breakdown^b^ (% high, moderate, inactive)	
	2013	2014	2013	2014	2013	2014	2013	2014	2013	2014	2013	2014
MS1	159 (99)	151 (92)	24.3	24.2	47	41	17.0 (16.0–18.1)	14.7 (13.5–15.9)	H: 23 M: 61 I: 16	H: 26 M: 62 I: 12	H: 40 M: 23 I: 37	H: 54 M: 18 I: 28
MS2	139 (89)	153 (95)	25.2	25.3	49	49	15.5 (14.5–16.5)	15.2 (14.2–16.3)	H: 32 M: 55 I: 13	H: 30 M: 61 I: 9	H: 50 M: 25 I: 25	H: 46 M: 27 I: 27
MS3	106 (54)	98 (63)	26.6	26.9	50	46	16.9 (15.5–18.3)	16.1 (14.6–17.6)	H: 14 M: 46 I: 40	H: 21 M: 37 I: 42	H: 25 M: 14 I: 61	H: 28 M: 25 I: 47
MS4	124 (67)	116 (59)	27.0	27.0	57	49	14.5 (13.4–15.8)	12.1 (11.0–13.3)	H: 34 M: 41 I: 25	H: 31 M: 48 I: 21	H: 45 M: 19 I: 36	H: 47 M: 22 I: 31
Total	528 (76)	518 (75)	25.6	25.9	50	46	16.0 (15.4–16.6)	14.6 (14.0–15.2)	H: 26 M: 52 I: 22	H: 27 M: 54 I: 19	H: 41 M: 21 I: 38	H: 45 M: 23 I: 32

^a^According to IPAQ scoring guidelines.

^b^According to CDC guidelines.

PSS: Perceived Stress Scale; IPAQ: International Physical Activity Questionnaire; PA: physical activity; HEPA: health-enhancing physical activity; CDC: Centers for Disease Control and Prevention.

### Associations between exercise and stress

Descriptive statistics of average stress levels across all medical students disclosed lower quartile (25%) PSS score cutoffs of 11, 11, 12, and 9 for MS1s, MS2s, MS3s, and MS4s, respectively. Upper quartile (75%) cutoffs for the same groupings were 22, 21, 22, and 19, respectively.

For each level increase in exercise intensity (e.g., going from moderate physical activity to HEPA), the odds of being in the highest quartile of stress compared to the other three quartiles were 30% less (95% CI 0.60–0.83; *p* < .001), holding all other variables constant (Table 2A). In this ordered logistic regression, the number of hours of sleep and gender were also statistically related to stress quartile (Table 2A). The predicted probability of belonging to the lowest stress quartile increased significantly as exercise intensity increased from inactive (0.21; 95% CI 0.17–0.24) to moderately active (0.27; 95% CI 0.24–0.29) to HEPA (0.34; 95% CI 0.30–0.38) (Table 2B). The inverse was also true: the probability of belonging to the highest stress quartile decreased as exercise intensity increased from inactive (0.30; 95% CI 0.26–0.35) to moderately active (0.23; 95% CI 0.21–0.26) to HEPA (0.18; 95% CI 0.15–0.21) (Table 2B).

**Table 2.** Results from the ordered logistic regression showing (A) odds ratios for belonging to the highest quartile of stress controlling for the listed independent variables and (B) predicted probabilities of belonging to the highest or lowest quartiles at stress at each intensity of exercise.^a^

A		
	OR (95% CI)	*p*-Value
IPAQ	0.70 (0.60–0.83)	<0.001
Sleep	0.80 (0.72–0.90)	<0.001
Female	1.61 (1.30–2.02)	<0.001
MS year	1.02 (0.92–1.12)	0.76

^a^Holding sleep, gender, and class year at their means.

### Physical activity

**Figure 1.**
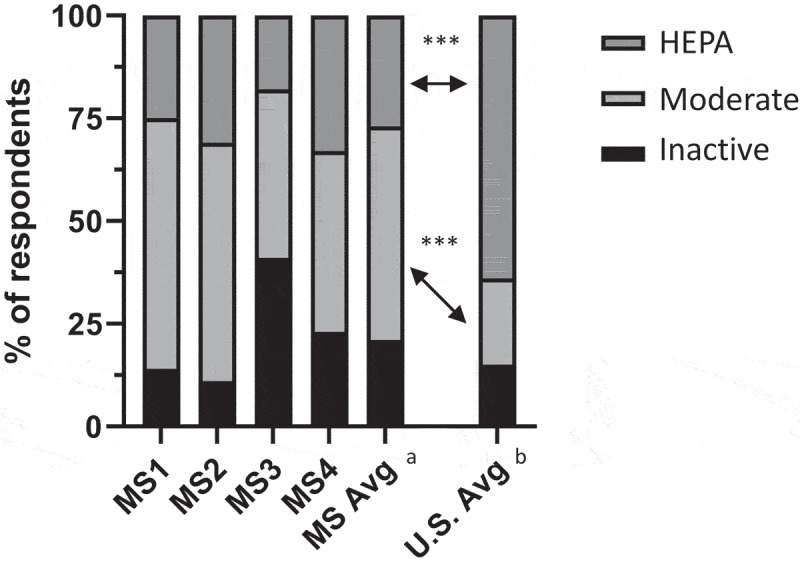
Breakdown of physical activity intensity according to the International Physical Activity Questionnaire among medical students and the age-adjusted national average.

^a^Average of all MS years across gender and both years of surveys. ^b^National average of all 18–39-year-olds in the U.S. between 2002 and 2004. Statistical comparisons are for odds ratios from chi-squared tests, with levels of significance as follows:   ***p < 0.001.

The distribution of medical student physical activity intensity in comparison to the age-adjusted U.S. average can be seen in Figure 1. Comparing the average of all students to the age-adjusted national average, we found that medical students were significantly less likely to be HEPA-active (27% vs. 64%; OR 0.21; 95% CI 0.12–0.37; *p* < .001) and correspondingly more likely to be moderately active (52% vs. 21%; OR 4.24; 95% CI 2.29–7.66; *p* < .001) (Figure 1). There was no statistical difference in the rates of inactivity between medical students and age-adjusted peers (21% vs. 15%; *p* = .36). These findings did not change when comparing both female and male medical students to the same-gendered national average (eFigure 1).

Applying the CDC guidelines for aerobic exercise to this study population showed that 65% of medical students were compliant with CDC aerobic exercise guidelines, which was not statistically different from the age-adjusted national average (65% vs. 58%, *p* = .16).

Averaging the 2 years of data, the proportionate breakdown of exercise intensity was statistically different among all years of training except between MS1s and MS2s, with large differences in exercise intensity between each level of training and the age-adjusted national average (eFigure 2). There were no statistical differences in the proportions of exercise intensity between genders except among MS2s (eFigure 2).

**Figure 2.**
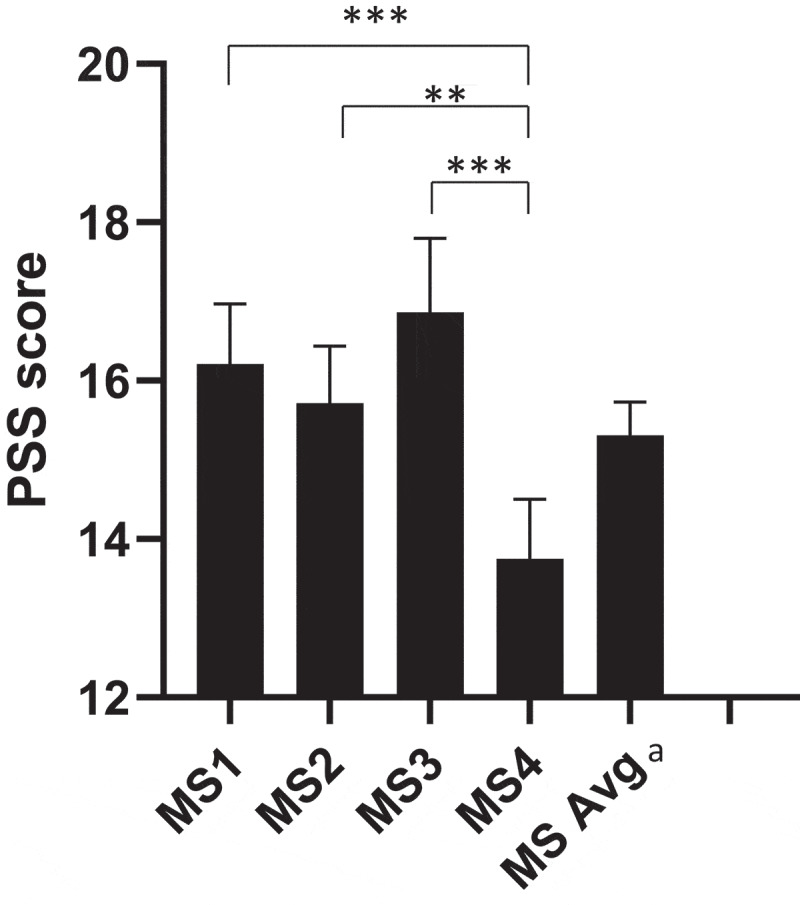
Perceived stress among medical students.

^a^Average of all MS years across both years of surveys. Error bars represent 95% confidence intervals. Statistical comparisons are for one-way ANOVA, with levels of significance as follows:  **p < 0.01, ***p < 0.001.

### Generalized perceived stress

A one-way ANOVA performed among levels of training showed that stress levels differed between years of medical school (*F*(3,1027) = 10.4; *p* < .001). A post-hoc analysis indicated that this difference was driven by MS1s vs. MS4s, MS2s vs. MS4s, and MS3 vs. MS4 (eFigure 3). Perceived stress levels were labile over the course of medical school, peaking during the third year and reaching the nadir in the fourth year (Figure 2). The ANOVAs excluded 15 out of 1,046 participants due to incomplete data.

## Discussion

To our knowledge, this is the first study to examine the relationship between optimal exercise intensity and perceived stress levels among U.S. medical students. The critical finding presented here is that the incremental intensity of exercise between moderate physical activity and HEPA was associated with a 26% increase in the predicted probability of being in the lowest stress quartile and a 22% decrease in the predicted probability of being in the highest stress quartile. Because the CDC guidelines [29] employed in previous studies only differentiate between compliance or noncompliance with aerobic exercise goals (and not between exercise intensities), they are unable to detect the benefits of optimal exercise intensity.

This investigation confirms prior studies [13,18,21,32,49] showing that medical students have comparable or better rates of compliance with the CDC aerobic exercise guidelines compared to an age-adjusted national average. Applying the IPAQ guidelines to these same medical students, however, revealed that they had 80% lower odds of engaging in HEPA compared to an age-adjusted national average. Furthermore, not achieving HEPA, even if moderate activity was achieved (which is compliant with the CDC guidelines), was associated with significantly higher stress levels and vice versa. The present results therefore build upon prior studies and indicate that a binary reporting system for exercise is insufficient to fully explore the relationship between exercise and distress. The associations revealed here support the use of a three-tiered exercise classification scheme that would allow examination of HEPA as a target for well-being interventions.

These findings indicate that future studies of medical trainee physical activity should track two specific indices of exercise activity that are absent from the CDC aerobic exercise guidelines. First, it may be important to track the number of days per week an individual is active to distinguish individuals who exercise 90 minutes 1 day per week from those who exercise 30 minutes 3 days per week. Spaced exercise throughout the week confers greater health benefits [33,34]. Second, it may be useful to differentiate between moderate (i.e., jogging for 20 minutes) and vigorous (i.e., sprinting for 20 minutes) levels of activity, the latter having a greater positive health impact [33,34].

Most interventions to improve medical trainee wellness have focused on duty hour restrictions and mindfulness-based programs, and very few have significantly improved rates of distress [16,24–26,50]. Exercise interventions have not been extensively explored, perhaps in part because physical activity levels have been thought to already be on par with the national average [13,18,21,32,49]. The present results suggest that it may be useful to more systematically examine the benefits of exercise intensity on the mental health of medical trainees, especially because the benefits of exercise interventions may promote health across more domains (e.g., physiological, psychosocial, and biochemical) [23] than more singular interventions. It is important to acknowledge that there are practical barriers that may prevent medical students from achieving HEPA guidelines. Foremost among these barriers are substantial time commitments and the physically taxing medical school curriculum, which likely explain why medical students tend to preferentially engage in moderate physical activity. In-room or around the block mini-bursts of exercise while studying or working are much more compatible with a busy schedule. While brief, less intense activity does have some benefit for the mental health of medical students, it is not sufficient to achieve HEPA and its associated additional mental health benefits. It may be helpful to encourage students to dedicate larger blocks of time (e.g., at least 60 minutes) to attaining the higher sustained heart rates of vigorous exercise required to achieve HEPA and even to incorporate such encouragement into the curriculum as has been done in other studies [51]. While it would be challenging to optimize physical activity among trainees, it may in fact be a practical and cost-effective strategy that could be compared to more traditional interventions such as work restrictions or mindfulness programs.

It is also important to note that women were more likely to belong to the highest quartile of stress after controlling for exercise intensity and sleep even though they were not less likely to achieve HEPA compared to men. This finding is consistent with prior studies that have found that women report higher stress levels on self-reported instruments than men [52], but requires further exploration in the setting of medical trainees’ physical activity.

By distinguishing among different intensities of activity, we have shown both that HEPA has a unique relation to perceived stress levels and that HEPA levels are not optimized among medical students. Future research should investigate whether interventions targeting HEPA can reverse or prevent outcomes such as burnout and depression. Specifically, a randomized controlled trial assigning medical students to an exercise regimen would be able to measure the exact mental health benefits of physical activity and determine causality. While psychological benefits have not been identified in prior studies of exercise [53], these benefits may not have been detected because earlier studies relied upon the CDC guidelines that did not differentiate between HEPA and less intense forms of exercise. It therefore may be more appropriate for future well-being interventions to target and measure HEPA separately from moderate physical activity, as opposed to grouping them together in the category of ‘compliant with CDC guidelines.’

### Limitations

This study had several limitations. First, a greater proportion of responders were first- and second-year students, so that our findings may be more applicable to the earlier stages of training. While the sample had lower proportions of third- and fourth-year students, similar results were seen across all years of training. Second, this two time-point study did not address directionality or causality of the relationship between HEPA and stress. As such, the present study could also be interpreted to suggest that medical students with lower perceived stress tend to exercise more and that enhancing coping mechanisms may result in other health-promoting behaviors. A randomized controlled trial affecting exercise and measuring stress would be best suited for research to establish causality. Third, this is a single-institution study. However, the 75% response rate is significantly higher than other similar survey-based investigations [13,21,32,54] and was conducted in Los Angeles where people are generally more physically active than in other parts of the country [55]. The high response rate makes this study powerful in its ability to capture a diverse and representative population of a single medical student body. Fourth, physical activity and stress were measured by self-report which introduces recall bias. Contemporaneous ratings of stress and exercise should be considered in future studies. Fifth, national data from the IPAQ have not been updated recently among a probability sample of the U.S. and are only stratified by age, making comparisons on the basis of education infeasible. Sixth, the data for this study were collected 7 to 8 years before their publication, potentially limiting the applicability of these findings. However, data from the CDC show that national levels of physical activity changed only minimally from 2013 to 2018 [42], suggesting that these findings would likely hold true at the time of publication.

## Conclusions

HEPA has important implications for the well-being of medical students because it exhibits a stronger inverse relationship with stress levels compared to lower intensities of exercise. While the CDC aerobic exercise guidelines suggest that medical students engage in comparable levels of physical activity to the national average, a more detailed analysis discloses that the odds of engaging in HEPA are 80% lower for medical students compared to the national average. This disparity highlights the need to promote longer bouts of high-intensity exercise (as opposed to mini-bursts) and to support students in achieving HEPA. Quantifying physical activity by differentiating between moderate physical activity and HEPA may help to make exercise an attractive and practical target for future wellness interventions.

## Supplementary Material

Supplemental Material

## Data Availability

The datasets used and analyzed during this study are available from the corresponding author upon reasonable request.

## References

[1] Koutsimani P, Montgomery A, Georganta K. The relationship between burnout, depression, and anxiety: a systematic review and meta-analysis. Front Psychol. 2019;10(MAR):284. 30918490 10.3389/fpsyg.2019.00284 PMC6424886

[2] Jackson ER, Shanafelt TD, Hasan O, et al. Burnout and alcohol abuse/dependence among U.S. medical students. Acad Med. 2016;91(9):1251–8. 26934693 10.1097/ACM.0000000000001138

[3] Dyrbye LN, Massie S, Eacker A, et al. Relationship between burnout and professional conduct and attitudes among US medical students. JAMA. 2010;304(11):1173–1180. 20841530 10.1001/jama.2010.1318

[4] Dyrbye LN, Thomas MR, V PD, et al. Burnout and serious thoughts of dropping out of medical school: a multi-institutional study. Acad Med. 2010;85(1):94–102. 20042833 10.1097/ACM.0b013e3181c46aad

[5] Dahlin M, Joneborg N, Runeson B. Performance-based self-esteem and burnout in a cross-sectional study of medical students. Med Teach. 2007;29(1):43–48. 17538833 10.1080/01421590601175309

[6] Dahlin ME, Runeson B. Burnout and psychiatric morbidity among medical students entering clinical training: a three year prospective questionnaire and interview-based study. BMC Med Educ. 2007;7(1):6. 17430583 10.1186/1472-6920-7-6 PMC1857695

[7] Pagnin D, de Queiroz V, Ytms C, et al. The relation between burnout and sleep disorders in medical students. Acad Psychiatry. 2014;38(4):438–444. 24683060 10.1007/s40596-014-0093-z

[8] Dyrbye LN, Thomas MR, Shanafelt TD. Systematic review of depression, anxiety, and other indicators of psychological distress among U.S. and Canadian medical students. Acad Med. 2006;81(4):354–373. 16565188 10.1097/00001888-200604000-00009

[9] Thomas MR, Dyrbye LN, Huntington JL, et al. How do distress and well-being relate to medical student empathy? A multicenter study. J Gen Intern Med. 2007;22(2):177–183. 17356983 10.1007/s11606-006-0039-6 PMC1824738

[10] Ishak W, Nikravesh R, Lederer S, et al. Burnout in medical students: a systematic review. Clin Teach. 2013;10(4):242–245. 23834570 10.1111/tct.12014

[11] Erschens R, Keifenheim KE, Herrmann-Werner A, et al. Professional burnout among medical students: systematic literature review and meta-analysis. Med Teach. 2019;41(2):172–183. 29656675 10.1080/0142159X.2018.1457213

[12] Thomas NK. Resident burnout. JAMA. 2004;292(23):2880–2889. 15598920 10.1001/jama.292.23.2880

[13] Olson SM, Odo NU, Duran AM, et al. Burnout and physical activity in minnesota internal medicine resident physicians. J Grad Med Educ. 2014;6(4):669–674. 26140116 10.4300/JGME-D-13-00396 PMC4477560

[14] Cecil J, McHale C, Hart J, et al. Behaviour and burnout in medical students. Med Educ Online. 2014;19:Published online 2014 10.3402/meo.v19.25209 PMC4145104 25160716

[15] Bugaj T, Cranz A, Junne F, et al. Psychosocial burden in medical students and specific prevention strategies. Ment Heal Prev. 2016;4(1):24–30

[16] Williams D, Tricomi G, Gupta J, et al. Efficacy of burnout interventions in the medical education pipeline. Acad Psychiatry. 2015;39(1):47–54. 25034955 10.1007/s40596-014-0197-5

[17] Wolf MR, Rosenstock JB. Inadequate sleep and exercise associated with burnout and depression among medical students. Acad Psychiatry. 2017;41(2):174–179. 26976402 10.1007/s40596-016-0526-y

[18] Frank E, Tong E, Lobelo F, et al. Physical activity levels and counseling practices of U.S. medical students. Med Sci Sport Exercise. 2008;40(3):413–421. 10.1249/MSS.0b013e31815ff399 18379201

[19] Macilwraith P, Bennett D. Burnout and physical activity in medical students. Ir Med J. 2018;111(3):707. 30376225

[20] Erschens R, Loda T, Herrmann-Werner A, et al. Behaviour-based functional and dysfunctional strategies of medical students to cope with burnout. Med Educ Online. 2018;23(1):1535738. 30371222 10.1080/10872981.2018.1535738 PMC6211255

[21] Dyrbye LN, Satele D, Shanafelt TD. Healthy exercise habits are associated with lower risk of burnout and higher quality of life among U.S. medical students. Acad Med. 2017;92(7):1006–1011. 28030419 10.1097/ACM.0000000000001540

[22] Steinmo S, Hagger-Johnson G, Shahab L. Bidirectional association between mental health and physical activity in older adults: Whitehall II prospective cohort study. Prev Med. 2014;66:74–79. 24945691 10.1016/j.ypmed.2014.06.005

[23] Griffiths A, Kouvonen A, Pentti J, et al. Association of physical activity with future mental health in older, mid-life and younger women. Eur J Public Health. 2014;24(5):813–818. Published online 2013. 24532567 10.1093/eurpub/ckt199 PMC4168042

[24] Busireddy KR, Miller JA, Ellison K, et al. Efficacy of interventions to reduce resident physician burnout: a systematic review. J Grad Med Educ. 2017;9(3):294–301. 28638506 10.4300/JGME-D-16-00372.1 PMC5476377

[25] West CP, Dyrbye LN, Erwin PJ, et al. Interventions to prevent and reduce physician burnout: a systematic review and meta-analysis. Lancet. 2016;388(10057):2272–2281. 27692469 10.1016/S0140-6736(16)31279-X

[26] Walsh AL, Lehmann S, Zabinski J, et al. Interventions to prevent and reduce burnout among undergraduate and graduate medical education trainees: a systematic review. Acad Psychiatry. 2019;43(4):386–395. 30710229 10.1007/s40596-019-01023-z

[27] Sun Y, Liu F, Wang Y, et al. Mindfulness improves health worker’s occupational burnout: the moderating effects of anxiety and depression. Int Arch Occup Environ Health. 2021;94(6):1297–1305. 33787983 10.1007/s00420-021-01685-z

[28] Hsieh HF, Liu Y, Hsu HT, et al. Relations between stress and depressive symptoms in psychiatric nurses: the mediating effects of sleep quality and occupational burnout. Int J Environ Res Public Health. 2021;18(14):7327. 34299778 10.3390/ijerph18147327 PMC8303432

[29] U.S. Department of Health and Human Services. Physical Activity Guidelines Advisory Committee. 2018. [Updated 2019 Feb 1; cited 2019 Mar 15. https://www.hhs.gov/fitness/be-active/physical-activity-guidelines-for-americans/index.html

[30] Konen JC, Fromn BS. Changes in personal health behaviors of medical students. Med Teach. 1992;14(4):321–325. 1293457 10.3109/01421599209018850

[31] Peterson DF, Degenhardt BF, Smith CM. Correlation between prior exercise and present health and fitness status of entering medical students. J Am Osteopath Assoc. 2003;103(8):361–366. 12956248

[32] Stanford FC, Durkin MW, Blair SN, et al. Determining levels of physical activity in attending physicians, resident and fellow physicians and medical students in the USA. Br J Sports Med. 2012;46(5):360–364. 22194220 10.1136/bjsports-2011-090299

[33] American Heart Association. American Heart Association Recommendations for Physical Activity in Adults. American Heart Association; [Updated 2018 Apr 18; cited 2019 Mar 15]. https://www.heart.org/en/healthy-living/fitness/fitness-basics/aha-recs-for-physical-activity-in-adults

[34] Piercy KL, Troiano RP, Ballard RM, et al. The Physical Activity Guidelines for Americans. JAMA. 2018;320(19):2020. 30418471 10.1001/jama.2018.14854 PMC9582631

[35] Craig CL, Marshall AL, Sjöström M, et al. International Physical Activity Questionnaire (IPAQ): 12-country reliability and validity. Med Sci Sport Exercise. 2003;35(8):1381–1395. 10.1249/01.MSS.0000078924.61453.FB 12900694

[36] Bauman A, Bull F, Chey T, et al. The International Prevalence Study on Physical Activity: results from 20 countries. Int J Behav Nutr Phys Act. 2009;6(1):21. 19335883 10.1186/1479-5868-6-21 PMC2674408

[37] Fogelholm M, Malmberg J, Suni J, et al. International Physical Activity Questionnaire. Med Sci Sport Exercise. 2006;38(4):753–760. 10.1249/01.mss.0000194075.16960.20 16679993

[38] Miilunpalo S. Evidence and theory based promotion of health-enhancing physical activity. Public Health Nutr. 2001;4(2b):725–728. 11683568 10.1079/phn2001163

[39] Cohen S, Hoberman HM. Positive events and social supports as buffers of life change stress. J Appl Soc Psychol. 1983;13(2):99–125.

[40] Cohen S, Janicki-Deverts D. Who’s stressed? Distributions of psychological stress in the usa in probability samples from 1983, 2006, and 2009. J Appl Soc Psychol. 2012. pp. 1320–1334; Published online.

[41] Jetté M, Sidney K, Blümchen G. Metabolic equivalents (METS) in exercise testing, exercise prescription, and evaluation of functional capacity. Clin Cardiol. 1990;13(8):555–565. 2204507 10.1002/clc.4960130809

[42] National Center for Health Statistics. Healthy People 2020 Data. [Updated 2021 Oct 27; cited 2021 Nov 9]. https://www.healthypeople.gov/2020/data-search

[43] Buysse DJ, Reynolds CF, Monk TH, et al. The Pittsburgh sleep quality index: a new instrument for psychiatric practice and research. Psychiatry Res. 1989;28(2):193–213. 2748771 10.1016/0165-1781(89)90047-4

[44] Janko MR, Smeds MR. Burnout, depression, perceived stress, and self-efficacy in vascular surgery trainees. J Vasc Surg. 2019;69(4):1233–1242. 30301691 10.1016/j.jvs.2018.07.034

[45] Tavolacci MP, Ladner J, Grigioni S, et al. Prevalence and association of perceived stress, substance use and behavioral addictions: a cross-sectional study among university students in France, 2009-2011. BMC Public Health. 2013;13(1). 10.1186/1471-2458-13-724 PMC3750571 23919651

[46] Charles LE, Slaven JE, Mnatsakanova A, et al. Association of perceived stress with sleep duration and sleep quality in police officers. Int J Emerg Ment Health. 2011;13(4):229–241. 22900457 PMC4681282

[47] Smeds MR, Janko MR, Allen S, et al. Burnout and its relationship with perceived stress, self-efficacy, depression, social support, and programmatic factors in general surgery residents. Am J Surg. 2020;219(6):907–912. 31307660 10.1016/j.amjsurg.2019.07.004

[48] Ozkaya G, Ercan I. Examining multiple comparison procedures according to error rate, power type and false discovery rate. J Mod Appl Stat Methods. 2012;11(2):348–360.

[49] Frank E, Galuska DA, Elon LK, et al. Personal and clinical exercise-related attitudes and behaviors of freshmen U.S. medical students. Res Q Exerc Sport. 2004;75(2):112–121. 15209329 10.1080/02701367.2004.10609142

[50] Ripp JA, Privitera MR, West C, et al. Well-being in graduate medical education. Acad Med. 2017;92(7):914–917. 28471780 10.1097/ACM.0000000000001735

[51] Worobetz A, Retief PJ, Loughran S, et al. A feasibility study of an exercise intervention to educate and promote health and well-being among medical students: the “MED-WELL” programme. BMC Med Educ. 2020;20(1):183. 32493427 10.1186/s12909-020-02097-2 PMC7271428

[52] Remes O, Brayne C, van der Linde R, et al. A systematic review of reviews on the prevalence of anxiety disorders in adult populations. Brain Behav. 2016;6(7):e00497. 27458547 10.1002/brb3.497 PMC4951626

[53] Weight CJ, Sellon JL, Lessard-Anderson CR, et al. Physical activity, quality of life, and burnout among physician trainees: the effect of a team-based, incentivized exercise program. Mayo Clin Proc. 2013; 88(12):435–1442. doi:10.1016/j.mayocp.2013.09.010; Published online. 24290117

[54] Wolf TM, Kissling GE. Changes in life-style characteristics, health, and mood of freshman medical students. J Med Educ. 1984;59(10):806–814. 6481777 10.1097/00001888-198410000-00005

[55] CDC - BRFSS. [Updated 2020 Aug 31; cited 2020 Sept 6]. https://www.cdc.gov/brfss

