# Supplementary material for: Relationship between exercise intensity and stress levels among U.S. medical students
Source: Med Educ Online. 2022 Jan 20;27(1):2027651. doi: 10.1080/10872981.2022.2027651 (PMC8786247; doi:10.1080/10872981.2022.2027651)
Supplement: Supplemental Material [file ZMEO_A_2027651_SM1631.docx]

^a^Percentage of this population achieving each exercise intensity

^b^Odds ratios calculated from chi-squared tests comparing each exercise intensity between the average of same-gendered medical students and the national average

**eFigure 1.** IPAQ exercise intensity by gender.

|  | **IPAQ**  **Inactive** | **IPAQ**  **Moderate** | **IPAQ**  **HEPA** |
| --- | --- | --- | --- |
| **MS Avg Females** | 21% | 57% | 22% |
| **MS Avg**  **Males** | 20% | 49% | 31% |
| **U.S. Avg**  **Females** | 18% | 26% | 56% |
| **U.S. Avg**  **Males** | 11% | 16% | 72% |
| **Females:**  **MS Avg v. U.S. avg** | OR 1.2;  95% CI, 0.6-2.4;  p=0.72 | **OR 3.8;**  **95% CI, 2.1-6.8;**  **p<0.001** | **OR 0.2;**  **95% CI, 0.1-0.4;**  **p<0.001** |
| **Males:**  **MS Avg v.**  **U.S. Avg** | OR 2.0;  95% CI, 0.9-4.3;  p=0.12 | **OR 5.0;**  **95% CI, 2.5-9.4; p<0.001** | **OR 0.17;**  **95% CI, 0.09-0.3;**  **p<0.001** |

**eFigure 2.** Chi-squared values comparing proportionate breakdown of exercise intensity among medical students and the U.S. average.^a^

^a^Each chi-squared has 2 degrees of freedom

^b^Average of all MS years across both years of surveys

^c^National average of those with professional degrees in the U.S. in 2006

^c^Chi-squared test comparing proportion of exercise intensities between genders within each level of training

|  | **MS1** | **MS2** | **MS3** | **MS4** | **U.S. Avg** | **Female v. male** |
| --- | --- | --- | --- | --- | --- | --- |
| **MS1** | -- | 1.1,  p=0.57 | **18.2, p<0.001** | **6.3, p=0.04** | **37.3,**  **p<0.001** | 1.7,  p=0.43 |
| **MS2** | -- | -- | **23.3,**  **p<0.001** | **6.2,**  **P=0.04** | **29.4,**  **p<0.001** | **6.3,**  **p=0.04** |
| **MS3** | -- | -- | -- | **9.5,**  **P=0.009** | **51.3,**  **p<0.001** | 3.3,  p=0.20 |
| **MS4** | -- | -- | -- | -- | **19.7,**  **p<0.001** | 0.5,  p=0.80 |
| **MS Avg** | -- | -- | -- | -- | **29.2, p<0.001** | 2.2,  p=0.34 |

**eFigure 3.** Post-hoc multiple comparisons from one-way ANOVAs comparing PSS scores among medical students.^a^

^a^Values in cells represent mean differences with 95% confidence intervals in parentheses. Statistically significant results are bolded.

|  | **MS1** | **MS2** | **MS3** |
| --- | --- | --- | --- |
| **MS1** | -- | -- | -- |
| **MS2** | 0.49  (-0.86-1.85) | -- | -- |
| **MS3** | -0.65  (-2.15-0.84) | -1.15  (-2.66-0.37) | -- |
| **MS4** | **1.97**  **(0.52-3.42)**  **p<0.001** | **1.97**  **(0.52-3.42)**  **p=0.003** | **3.11**  **(1.53-4.69)**  **p<0.001** |
